# Charcot-Marie-Tooth disease type 2CC due to *NEFH* variants causes a progressive, non-length-dependent, motor-predominant phenotype

**DOI:** 10.1136/jnnp-2021-327186

**Published:** 2021-09-13

**Authors:** Menelaos Pipis, Andrea Cortese, James M Polke, Roy Poh, Jana Vandrovcova, Matilde Laura, Mariola Skorupinska, Arnaud Jacquier, Raul Juntas-Morales, Philippe Latour, Philippe Petiot, Guilhem Sole, Yves Fromes, Sachit Shah, Julian Blake, Byung-Ok Choi, Ki Wha Chung, Tanya Stojkovic, Alexander M Rossor, Mary M Reilly

**Affiliations:** 1 Centre for Neuromuscular Diseases, UCL Queen Square Institute of Neurology, London, UK; 2 Department of Brain and Behavioral Sciences, University of Pavia, Pavia, Italy; 3 Institut NeuroMyoGène, CNRS UMR5310, INSERM U1217, Universite de Lyon, Lyon, France; 4 Clinique du Motoneurone et Pathologies Neuromusculaires, CHRU de Montpellier, Montpellier, France; 5 Centre de Biologie et Pathologie Est, Hospices Civils de Lyon, Lyon, France; 6 Neurologie et Explorations Fonctionnelles Neurologiques, Centre de Référence Maladies Neuromusculaires, Hospices Civils de Lyon, Lyon, France; 7 Centre de Référence des Maladies Neuromusculaires, CHU Bordeaux GH Pellegrin, Bordeaux, France; 8 Institut de Myologie, Laboratoire RMN, Hôpital Pitié-Salpêtrière, Paris, France; 9 Neuroradiological Academic Unit, UCL Queen Square Institute of Neurology, London, UK; 10 Department of Clinical Neurophysiology, Norfolk and Norwich University Hospital, Norfolk, UK; 11 Department of Neurology, Samsung Medical Center, Sungkyunkwan University School of Medicine, Seoul, South Korea; 12 Department of Biological Sciences, Kongju National University, Gongju, South Korea; 13 AP-HP, Reference Center for Neuromuscular Disorders, University Hospital Pitié Salpêtrière, Paris, France; 14 Centre de Recherche en Myologie, Inserm UMRS974, Sorbonne Universite, Paris, France

**Keywords:** HMSN (Charcot-Marie-Tooth), neurogenetics, neuromuscular, neuropathy

## Abstract

**Objective:**

Neurofilaments are the major scaffolding proteins for the neuronal cytoskeleton, and variants in *NEFH* have recently been described to cause axonal Charcot-Marie-Tooth disease type 2CC (CMT2CC).

**Methods:**

In this large observational study, we present phenotype–genotype correlations on 30 affected and 3 asymptomatic mutation carriers from eight families.

**Results:**

The majority of patients presented in adulthood with motor-predominant and lower limb-predominant symptoms and the average age of onset was 31.0±15.1 years. A prominent feature was the development of proximal weakness early in the course of the disease. The disease progressed rapidly, unlike other Charcot-Marie-Tooth disease (CMT) subtypes, and half of the patients (53%) needed to use a wheelchair on average 24.1 years after symptom onset. Furthermore, 40% of patients had evidence of early ankle plantarflexion weakness, a feature which is observed in only a handful of CMT subtypes. Neurophysiological studies and MRI of the lower limbs confirmed the presence of a non-length-dependent neuropathy in the majority of patients.

All families harboured heterozygous frameshift variants in the last exon of *NEFH*, resulting in a reading frameshift to an alternate open reading frame and the translation of approximately 42 additional amino acids from the 3' untranslated region (3′-UTR).

**Conclusions:**

This phenotype–genotype study highlights the unusual phenotype of CMT2CC, which is more akin to spinal muscular atrophy rather than classic CMT. Furthermore, the study will enable more informative discussions on the natural history of the disease and will aid in *NEFH* variant interpretation in the context of the disease’s unique molecular genetics.

## Introduction

Neurofilaments (NFs) are intermediate filaments with an average diameter of 10 nm that are almost exclusively expressed in neurons.^1^ The majority of them are the NF triplet subunit proteins that are classified according to their gel electrophoresis-derived molecular weight into heavy (NEFH, 205 kDa), medium (NEFM, 160 kDa) and light (NEFL, 68 kDa) subunits. They assemble as heteropolymers to become the major scaffolding component of the mature neuronal cytoskeleton in both peripheral and central neurons, and they primarily have a structural role in determining axonal calibre.^1 2^ Peripherin and α-internexin are other NF components of the neuronal cytoskeleton in the peripheral nervous system (PNS) and central nervous system (CNS), respectively.^1 3^

Experimental studies on NFs over the past four decades have provided us with a wealth of knowledge on their expression, structure, assembly, transport and function. All three NF triplet proteins share a common structure consisting of three components: a head domain (non-helical amino terminus), a central alpha-helical rod domain and a tail domain (non-helical carboxyl terminus).^4^

The architectural assembly of NFs relies, among others, on two important factors: first, the line-up and appropriate association of conserved alpha-helical rod domains on opposing NF molecules, and second, the inherent property of NFs to form cross-bridges between them.^1 3^ Immunofluorescence thin-section studies from healthy mammalian neurons show NEFH subunits primarily located at interfilament cross-bridges and being sparse or absent from neuronal cell bodies. This suggests a selective role of NEFH in the formation of interfilament cross-bridges, a function which is most prevalent in mature axons.^1 5^

The tail domains of NEFM and NEFH contain multiple repeat motifs of lysine-serine-proline amino acids (KSP repeats) which act as phosphorylation sites with important roles in interfilament cross-bridging and axonal calibre expansion and stabilisation.^3 6 7^ Various pieces of evidence illustrate the important roles the NEFH carboxyl terminus plays at different stages in neuronal development and especially in axonal radial expansion and maintenance.^6 8^

There is evidence that accumulation of NFs either as stand-alone aggregates or as part of neurofibrillary tangles or Lewy bodies plays a role in neurodegenerative conditions of the CNS such as amyotrophic lateral sclerosis (ALS), Alzheimer’s disease and Parkinson’s disease and of the PNS such as Charcot-Marie-Tooth disease (CMT), and it is not a surprise that they are explored as potential biomarkers for these diseases.^1 9^ Furthermore, single-nucleotide polymorphisms and deletions in the KSP repeat motifs of the NEFH tail domain have specifically been associated with an increased risk of ALS.^10 11^

In CMT, heterozygous mutations in *NEFL* cause autosomal dominant CMT1 or CMT2,^12^ whereas biallelic mutations cause an early onset and severe axonal neuropathy with associated slow conduction velocities.^13–15^ Overall the neuropathy associated with *NEFL* mutations is axonal and, to date, there is no convincing evidence that primary demyelination contributes to the pathogenesis of *NEFL*-related CMT.^16 17^ The reduced motor conduction velocities reported in some patients are usually secondary to reduced diameter of the axons confirmed from sural nerve biopsies or skin biopsies containing dermal myelinated nerve fibres; in addition, these biopsies do not show features of primary demyelination.^14 15 18^ More recently, heterozygous pathogenic frameshift variants in the last exon of *NEFH* have been reported to cause CMT2^19–21^; an *NEFM-*related CMT phenotype has not been reported so far.

The aim of this observational cohort study was to characterise the detailed phenotype and progression of Charcot-Marie-Tooth disease type 2CC (CMT2CC) due to mutations in the *NEFH* gene in 30 affected and three asymptomatic mutation carriers from eight unrelated families. Furthermore, the study highlights the unique genetic pathomechanism in this form of CMT, which is always associated with frameshift mutations in the last exon of the gene.

## Materials and methods

### Patient recruitment

Patients included in this study were recruited to the respective research protocols of the assessing clinical centres. Patients were reviewed at least once in a clinical setting, and in cases of multiple visits, clinical data from the most recent visit were included in the cross-sectional correlations presented in this study.

### Clinical and neurophysiological assessment

All patients with detailed phenotypical data presented in this study were assessed by the principal investigators (MMR, AMR, JB, AC, TS, B-OC and KWC). Motor examination scores were assigned according to the Medical Research Council motor grading scale (0–5). Where available, the Charcot-Marie-Tooth Examination Score V.2.0 (CMTESv2) was included. (This is a composite score based on patients’ symptoms and examination findings; a higher score indicates a greater degree of disability). Neurophysiological assessments included nerve conduction studies and electromyography (EMG) with concentric needle electrodes performed according to local protocols and normative values.

### Muscle MRI

MRI with neuromuscular protocols was obtained from four patients, and this included T1-weighted and short tau inversion–recovery (STIR) axial images of the thighs and calves. Furthermore, whole body coronal and axial T1-weighted magnetic resonance images were acquired from patient FR2-II.1.

### Molecular genetic analysis

Genomic DNA was extracted according to standard procedures. Whole or mini-exome sequencing was performed on the probands from five families using the Illumina HiSeq2500 sequencing platform (UK1, UK4 and CN1) or the Illumina NextSeq500 platform (FR1 and FR2). In-house CMT gene-specific panels were applied on the probands of the remaining three families (UK2, UK3 and FR3). The Genome Analysis Toolkit software was used for sequence alignment and variant calling. All individuals who had blood available for genotyping are indicated in table 1 and the family pedigrees; the *NEFH* genotype was confirmed through Sanger sequencing with primers that have been designed in-house using the NCBI’s Primer Basic Local Alignment Search Tool and SNPCheck.

**Table 1:** *NEFH* genotypes

No	Family identifier/ancestry	Family members with available phenotype/(genotype) data	*NEFH* variant details		Reference
			SNV/indel	Amino acid change	
1	UK1	4 / (3)	c.3010_3011delGA	p.(Asp1004Glnfs*58)	Rebelo *et al* 20
2	UK2	2 / (1)	c.3010_3011delGA	p.(Asp1004Glnfs*58)	This study
3	UK3	1 / (1)	c.3010_3011delGA	p.(Asp1004Glnfs*58)	This study
4	UK4	11 / (7)	c.3017dup	p.(Pro1007Alafs*56)	This study
5	FR1	11 / (11)	c.3008_3009delAA	p.(Lys1003Argfs*59)	Jacquier *et al* 19
6	FR2	1 / (1)	c.3043_3044delAA	p.(Lys1015Glyfs*47)	Jacquier *et al* 19
7	FR3	1 / (1)	c.3057dup	p.(Lys1020Glufs*43)	This study
8	CN1	2 / (2)	c.3015_3027dup CAAGCCTCCAGAG (de-novo)	p.(Lys1010Glnfs*57)	Nam *et al., 2017* 21

indel, insertion or deletion; SNV, single-nucleotide variation.

### Statistical analysis

Due to the small sample size, descriptive statistics were used for any cross-sectional correlations with the exception of Fisher’s exact test, which was used where appropriate. In the manuscript, continuous data are represented by values of the mean±SD.

## Results

**Figure 1:**
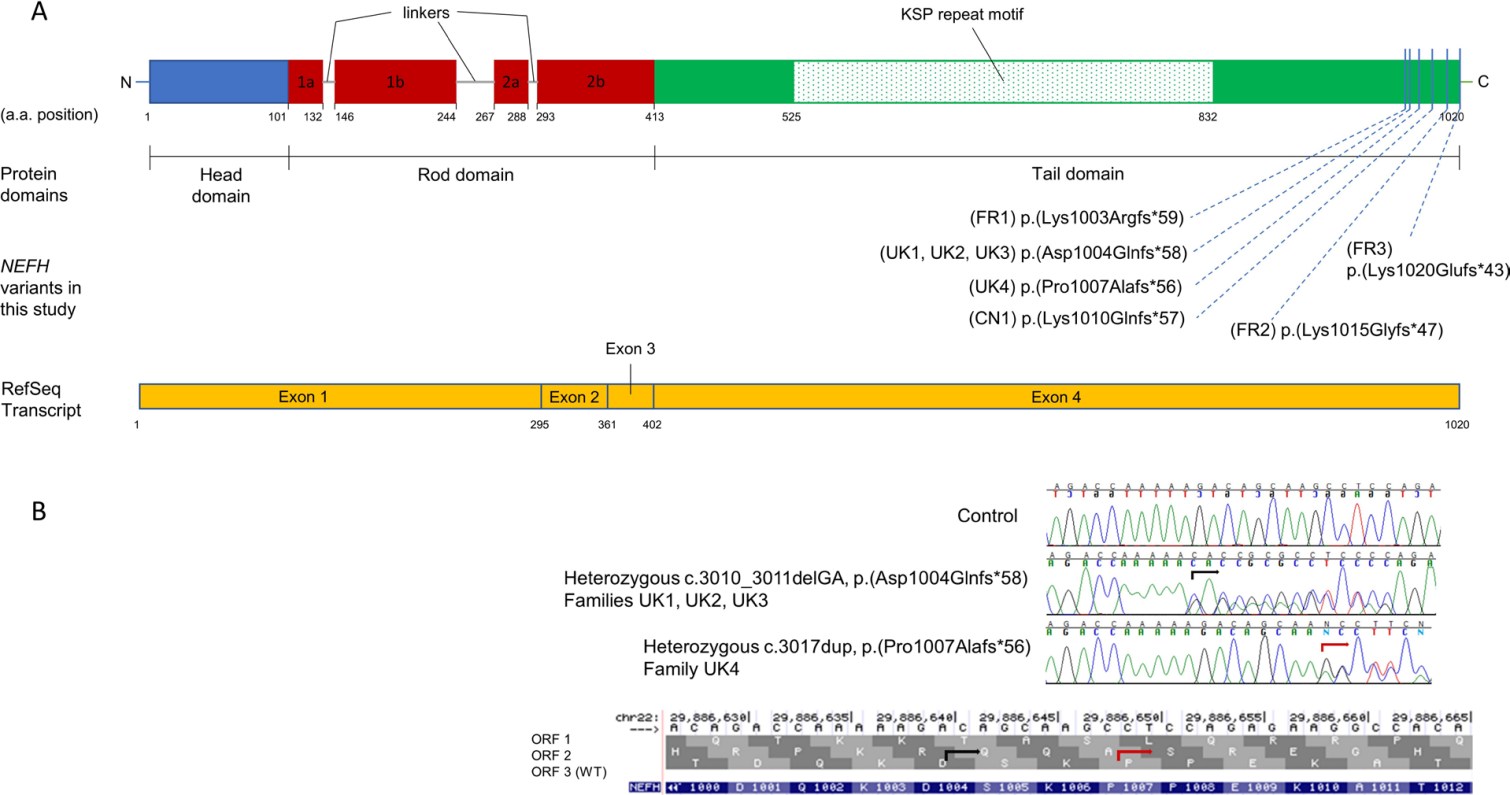
Topology of *NEFH* variants in relation to the protein domains and transcript and the frameshift to an alternate open reading frame. (A, panel above) Schematic representation of the NEFH protein and the corresponding RefSeq transcript. All variants in this study were in the tail domain of *NEFH*, which is coded for by exon 4. (B, panel below) Electropherogram obtained from the Sanger confirmation of two of the *NEFH* variants and illustration how both frameshift variants, c.3010_3011delGA (black arrow) and c.3017dup (red arrow), change the wild-type ORF3 to the alternate ORF2. KSP, lysine-serine-proline; ORF, open reading frame.

**Figure 2:**
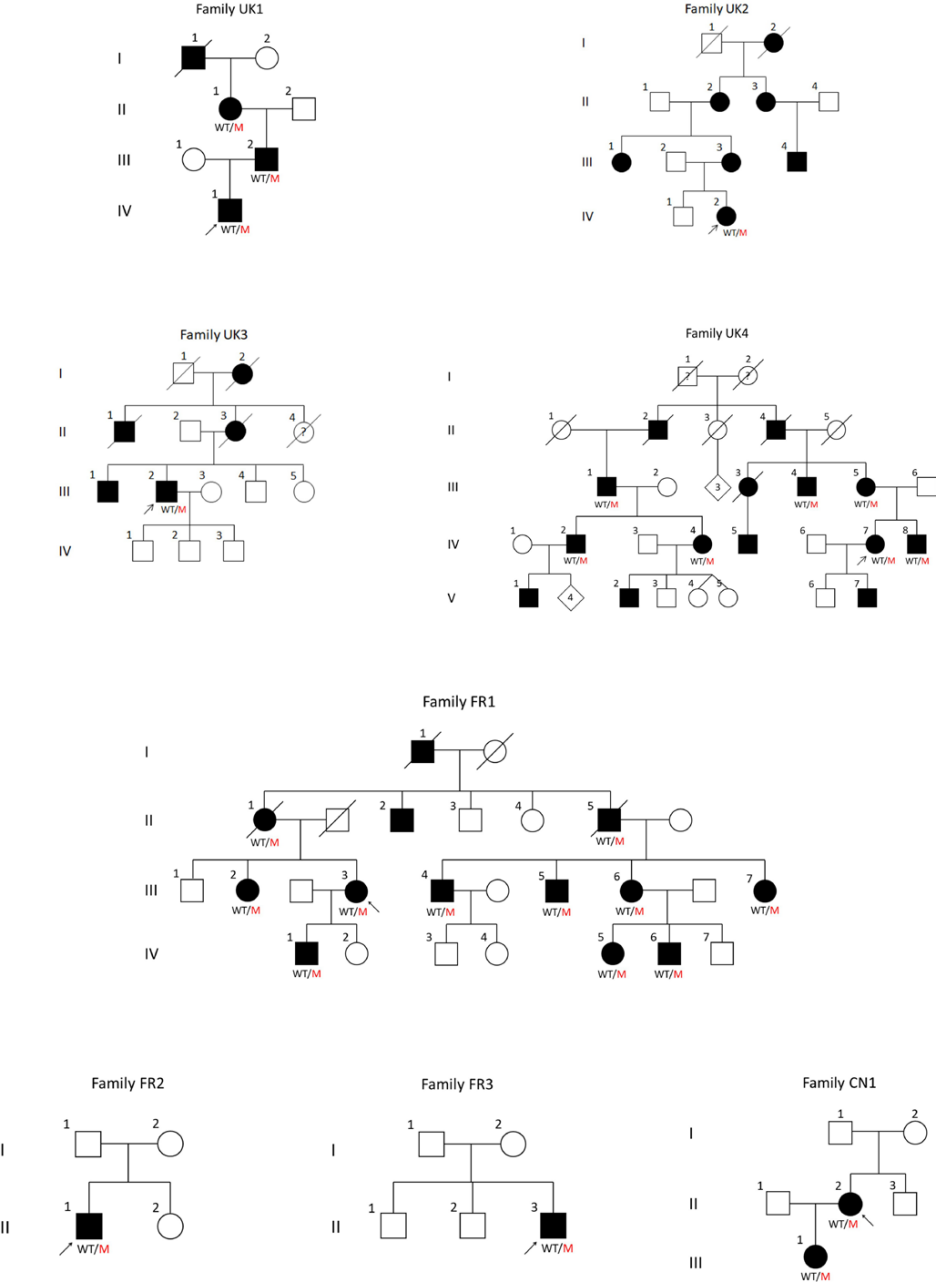
Pedigrees of newly identified and previously published families with Charcot-Marie-Tooth disease type 2CC reported in this study. All pedigrees are numbered as they are referenced in the manuscript and tables. Arrows indicate the proband from each family. Filled symbols denote affected individuals; squares are men and circles are women. In cases where the genotype was available, it is indicated under the corresponding symbol as WT/M.

In this genotype–phenotype study, we present phenotypical data on 33 patients from eight families with CMT2CC due to heterozygous pathogenic frameshift *NEFH* variants (table 1 and figure 1). Families UK1,^20^ FR1, FR2^19^ and CN1^21^ have been reported previously with limited clinical information, and the remaining four new families are reported here. The collated phenotypical data and pedigrees from all eight families are presented in the online supplemental table and figure 2, respectively, and further details of the four new families are in the online supplemental material document.

10.1136/jnnp-2021-327186.supp1 Supplementary data

10.1136/jnnp-2021-327186.supp2 Supplementary data

### Atypical clinical presentation and rapid progression characteristic of CMT2CC

**Figure 3:**
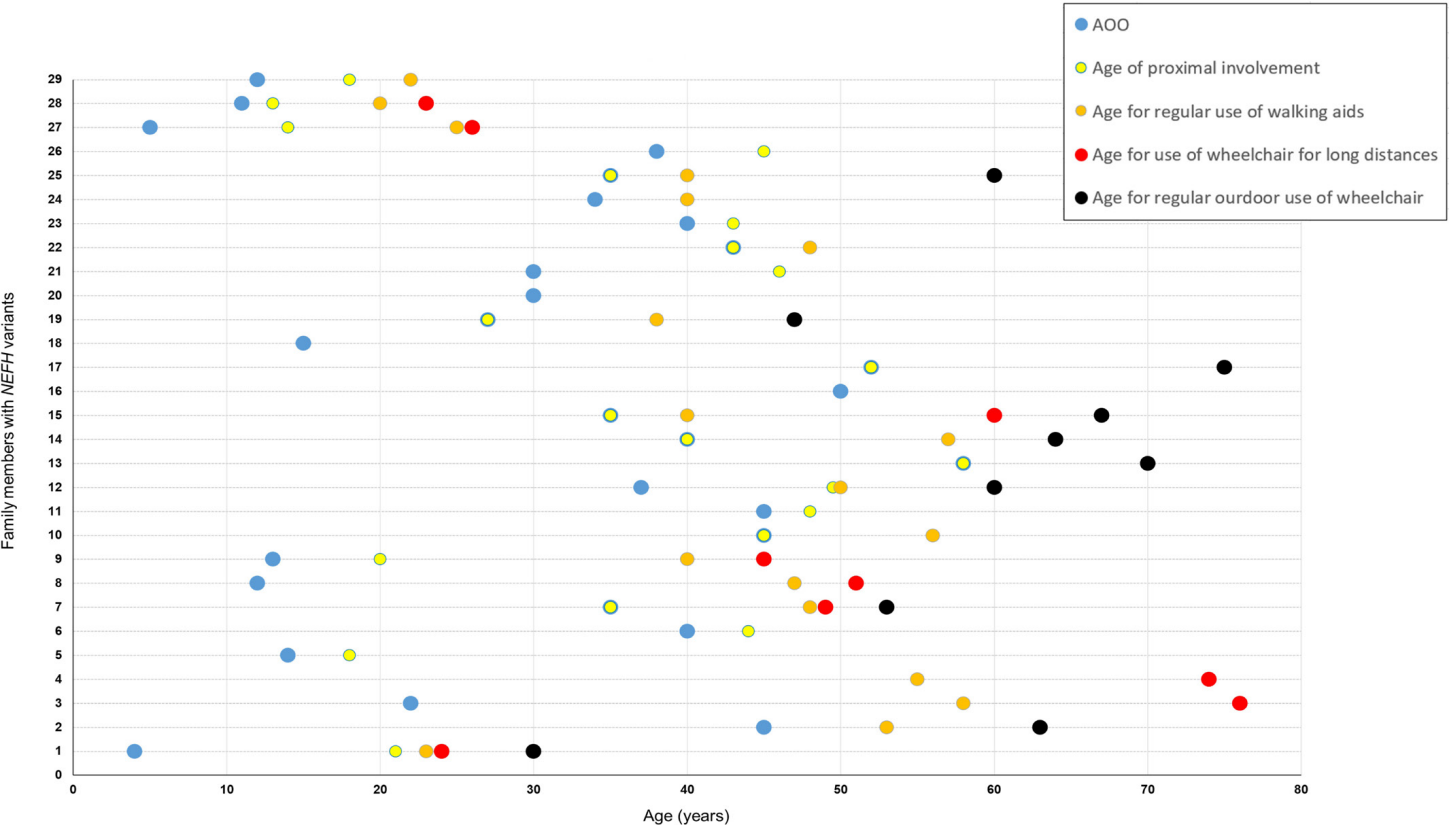
Natural progression of disability in symptomatic patients with CMT2CC in this study. Each horizontal dotted line and the corresponding timepoints on that line represent 1 of 29 symptomatic patients from this CMT2CC cohort on which there are available clinical data (individual UK2–III.3 has not been included as there were no available data on age of onset and age when walking aids were first needed). On the Y-axis, family UK1 is represented by 1–4 (in the following order: IV.1, III.2, II.1, I.1); UK2–IV.2 is represented by 5; UK3–III.2 is represented by 6; family UK4 is represented by 7–17 (in the following order: IV.7, IV.2, IV.4, IV.5, IV.8, III.1, III.3, III.4, III.5, II.2, II.4); family FR1 is represented by 18–26 (in the following order: IV.1, III.3, III.2, III.4, III.5, III.6, III.7, II.1, II.5); FR2–II.1 is represented by 27; FR3–II.3 is represented by 28; and CN1–II.2 is represented by 29. The blue circle represents the AOO of symptoms; the yellow circle represents the age of initial proximal muscle involvement; the orange circle represents the age when walking aids where first needed; the red circle represents the age when a wheelchair was first needed for long distances; and the black circle represents the age when a wheelchair was first needed on a full-time basis. For patients who had features suggesting proximal weakness as the onset of symptoms, the blue and yellow circles coincide. AOO, age of onset; CMT2CC, Charcot-Marie-Tooth disease type 2CC.

Available clinical information for 33 patients (28 seen in our centres and 5 by history), of which 30 were symptomatic, indicated that the majority first presented with neuropathy symptoms in mid-adulthood with an average age of onset of 31.0±15.1 years (SD); eight patients had onset of the condition in childhood or teenage years. A common and important clinical feature of the phenotype, which has functional implications on daily life, was the development of proximal lower limb (LL) weakness usually in the form of difficulties climbing stairs, walking uphill or standing from sitting. This became evident in 70% of patients (21/30) early in the course of the disease with an average time from the onset of symptoms to proximal involvement of 4.4±5.4 years (SD). Nine patients presented with features suggesting proximal involvement, and in all, onset of symptoms was on or after the age of 20 years. A presentation with proximal weakness was significantly more likely to occur in adult-onset (onset on or after 20 years) compared with childhood-onset (defined as onset before 20 years) cases (60% (9/15) vs 0% (0/6), Fisher’s exact test p=0.019). Furthermore, approximately half of symptomatic patients in this study (16/30, 53%) reverted to using a wheelchair or mobility scooter at some point in their lifetime, either for travelling long distances or on a permanent basis. Fourteen of these 16 patients required walking aids (eg, stick) before the age of 65 years, and the average time between onset of symptoms and any use of a wheelchair or mobility scooter was 24.1±10.9 years (SD). A summative graphical representation of the disease progression for each patient is shown in figure 3. Three patients seen in our centres at the ages of 6, 17 and 23 years were asymptomatic. However, in two of these individuals (FR1-IV.5 and FR1-IV.6), there was neurophysiological evidence of an axonal neuropathy, with reduced sensory responses in the LL and evidence of distal LL chronic denervation on EMG testing.

Interestingly, 40% of the symptomatic patients seen in our centres (10/25) showed evidence of early ankle plantarflexion weakness compared with ankle dorsiflexion, and this was obvious either by history (such as inability to stand on toes) and/or by examination. However, this phenotypical feature did not have a statistically significant association with the time from onset of symptoms to first use of a wheelchair or mobility scooter (two-tailed Mann-Whitney U value of 19, not significant). Fifty-two per cent of patients (13/25) had some form of foot deformity, and patients from two of the eight families (UK1 and FR1) displayed subtle upper motor neuron features on examination in the form of brisk reflexes.

Thirteen individuals had CMTESv2 data at a specific age of examination (online supplemental table). A two-tailed Spearman’s rank correlation coefficient showed no significant correlation (Spearman’s r=0.48, p=0.094) between the CMTESv2 and the disease duration (age at examination minus age of onset).

In families UK1 and FR1, there was intrafamilial variability with successive generations of affected individuals showing an earlier onset of the disease (FR1) and/or a more severe phenotype as illustrated by the level of disability accrued and age at which walking aids and wheelchair use were needed (UK1).

### Neurophysiology

All patients who had nerve conduction studies (20/33) showed evidence of a motor-predominant sensorimotor axonal neuropathy (table 2). LL motor and sensory responses were universally reduced or absent, whereas upper limb (UL) motor responses were reduced only in two patients over the age of 65 years. The neurophysiological severity of the neuropathy varied between patients, irrespective of their age at which the studies were done.

**Table 2:** Summary of neurophysiological studies

Family no	Patient	Age (years) at assessment	Median motor (APB)		Ulnar motor (FDIO)		Peroneal motor (EDB)		Tibial motor (AH)		Median sensory		Ulnar sensory		Radial sensory		Sural sensory		EMG/comments
			Amp (mV)	CV (m/s)	Amp (mV)	CV (m/s)	Amp (mV)	CV (m/s)	Amp (mV)	CV (m/s)	Amp (µV)	CV (m/s)	Amp (µV)	CV (m/s)	Amp (µV)	CV (m/s)	Amp (µV)	CV (m/s)	
UK1	IV.1	24	10.7	46	10.4 (ADM)	54	1.9	35	1.2	–	6	56	3	52	20	56	A	NA	Proximal and distal, non-length-dependent chronic denervation in the UL and LL; some equivocal myopathic change in some muscles
	III.2	56	6.7	54	12.1 (ADM)	51	0.5	41	–	–	3	51	1	52	11	60	A	NA	Proximal and distal non-length-dependent chronic denervation
	II.1	78	1.3	39	5.9 (ADM)	54	A	NA	A	NA	A	NA	A	NA	A	NA	A	NA	Proximal and distal non-length-dependent chronic denervation
UK2	IV.2	19	10.7	44	8.5 (ADM)	51	2	43	1.1	–	18	50	7	60	31	64	5	43	Limited EMG study (tibialis anterior) was unremarkable
UK3	III.2	44	6.5	42	7.1 (ADM)	46	1.9	41	1.5	–	2	52	A	NA	6	52	A	NA	Prominent chronic denervation in UL and LL, largely length-dependent
UK4	IV.7	53	6.7	50	8.4 (ADM)	55	2.3	33	0.9	NA	10	52	3	53	19	57	2	36	Prominent chronic non-length-dependent denervation, similar distally and proximally, with features of acute denervation
	IV.8	50	9.8	50	7.9 (ADM)	59	2	43	1	NA	5	57	4	56	14	62	A	NA	Chronic denervation, not convincingly length-dependent and similar distally and proximally; with features of acute denervation
FR1	II.5	68	12.1	53.8	–	–	0.12	31.1	–	–	A	NA	A	NA	–	–	A	NA	Non-length-dependent chronic denervation in the UL and LL
	III.2	55	7.4	54.3	–	–	5	43.2	–	–	13.7	40	–	–	–	–	4.5	39	Non-length-dependent chronic denervation in the UL and LL
	III.3	51	10.6	65	7.6	52	2.4	31	–	–	12	38	5	45	11	44	2	31	Non-length-dependent chronic denervation in the UL and LL
	III.4	50	8.2	50	–	–	–	–	–	–	A	NA	A	NA	–	–	A	NA	Non-length-dependent chronic denervation in the LL
	III.5	49	6.4	45.1	8.5	49.8	A	NA	–	–	A	NA	A	NA	–	–	A	NA	Features consistent with chronic denervation distally in the LL
	III.6	46	7.8	44.2	11.4	50	2.4	30.8	–	–	6.3	38	3.3	43	–	–	A	NA	Non-length-dependent chronic denervation in the LL
	III.7	44	3.4	51.1	9	43.6	4.1	41.1	–	–	4.2	39	7.7	41	–	–	4.5	33	Non-length-dependent chronic denervation in the LL
	IV.1	23	13.07	53	11.81	56	0.77	37	–	–	47.8	37	5.4	39	14.3	38	9.6	25	Features consistent with chronic denervation distally in the LL
	IV.5	23	8.2	55.2	–	–	5.8	39.1	–	–	10.1	–	10.3	48	–	–	A	NA	Features consistent with chronic denervation distally in the LL
	IV.6	17	8	50.2	–	–	6.5	38.5	–	–	11.5	–	14.7	47	–	–	4.1	40	Features consistent with chronic denervation distally in the LL
FR2	II.1	23	5.77	54	6.8	59	1.35	34	–	–	3.5	40	A	NA	5	40	A	NA	Non-length-dependent chronic denervation
FR3	II.3	24	10	45.1	6.7	46	2	44.3	1.2	38.6	2.1	41.9	–	–	4.8	40.7	–	–	Chronic denervation, equally prominent distally and proximally, with features of acute denervation
CN1	II.2	28	19.4	44.7	12.6	48.3	1.2	30.6	7.1	39.8	10	41.3	8	39.3	7.7	50	6	34.6	Non-length-dependent chronic denervation

All motor amplitudes are responses to distal stimulation (wrist and ankle).

–, not available; A, absent; ADM, abductor digiti minimi; AH, abductor hallucis; Amp, amplitude; APB, abductor pollicis brevis; CV, conduction velocity; EDB, extensor digitorum brevis; EMG, electromyography; FDIO, first dorsal interosseous; LL, lower limb; NA, not applicable; UL, upper limb.

EMG revealed a clear pattern of non-length-dependent denervation in the LL with/without UL involvement in 70% of patients (14/20). Chronic denervation changes were evident, as expected, with reduced recruitment of motor units that were usually polyphasic, with or without an increased duration. In most cases, large motor units were observed firing at high rates with a reduced interference pattern. In 3 of the 20 patients sampled with EMG, there was also evidence of some active denervation in the biceps (UK4-IV.8 and FR3-II.3) and the tibialis anterior (UK4-IV.7 and FR3-II.3) in the form of fibrillations and positive sharp waves.

### Muscle and CNS MRI

**Figure 4:**
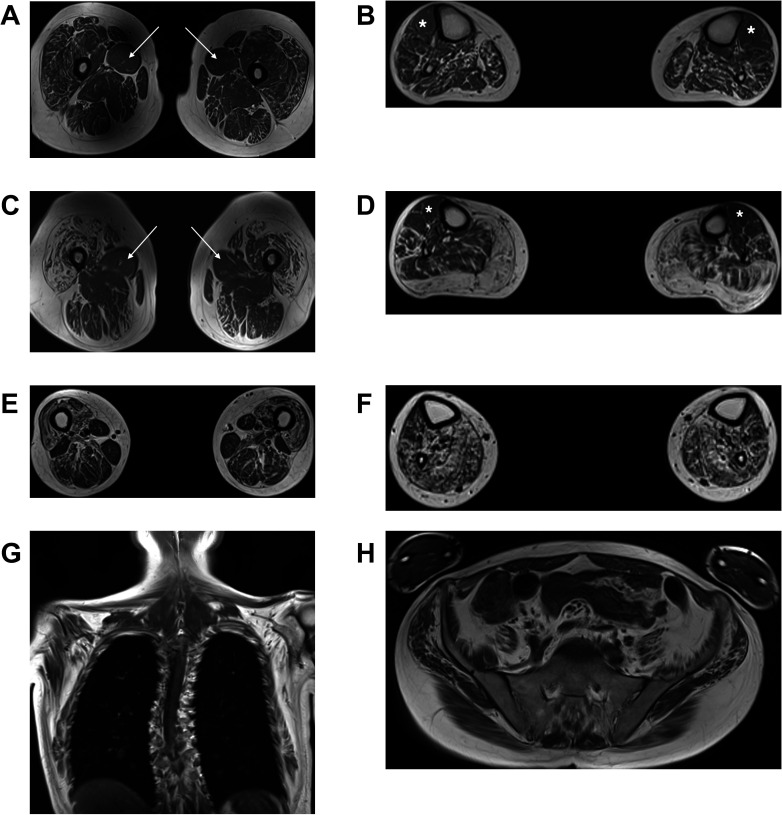
Muscle MR images in patients with Charcot-Marie-Tooth disease type 2CC. The panels show T1-weighted MR images of the thighs and calves in individuals UK1-IV.1 (A, B), UK4-IV.7 (C, D) and FR2-II.1 (E–H). In all three patients, the degree of intramuscular fat accumulation in the thighs is similar to that in the calves. In individual UK1-IV.1 at the thigh level (A), there is streaky fatty change particularly involving vastus lateralis, vastus intermedius and rectus femoris, with conspicuous sparing and relative hypertrophy of adductor longus (white arrows). In the calves (B), there is generalised intramuscular fatty change, more pronounced in the posterior compartment, with relative sparing of the tibialis anterior (white asterisks). In individual UK4-IV.7 at the thigh level (C), there is severe fatty degeneration of the quadriceps with small ‘islands’ of preserved motor units indicating advanced neurogenic amyotrophy. Again, there is relative sparing of the adductor longus (white arrows). In the calves (D), there is almost complete fatty replacement of the gastrocnemius, with less marked streaky fatty changes elsewhere and relative sparing of the tibialis anterior (white asterisks) and the extensor digitorum longus. In individual FR2-II.1, who clinically and radiologically is the most severely affected of the three patients, the thigh images (E) show severe fatty change of the quadriceps, with less marked changes involving the posterior compartment. In the calves (F), there is severe fatty change affecting the posterior compartment muscles, with slightly less severe involvement of the tibialis anterior and the extensor digitorum longus. Whole body T1-weighted MR images acquired in this individual (G, H) also demonstrate widespread symmetric involvement of the shoulder girdle, chest wall and pelvic muscles. MR, magnetic resonance.

Four patients from four families underwent muscle MRI of the calves and thighs (UK1-IV.1, UK4-IV.7, FR2-II.1 and CN1-II.1^21^). All cases displayed a clear pattern of non-length-dependent intramuscular fat accumulation which was symmetrical, with the degree of thigh muscle involvement being similar to that of the calves (figure 4). More specifically, at the thigh level, there was pronounced intramuscular fat accumulation in the anterior compartment muscles compared with the posterior

compartment, with relative sparing and hypertrophy of the adductor longus in three cases (UK1-IV.1, UK4-IV.7 and CN1-II.1). At the calf level, there were generalised intramuscular fatty changes, with more marked involvement of the posterior compartment muscles. Strikingly, all cases showed relative sparing of the tibialis anterior and the extensor digitorum longus. The whole body MRI (FR2-II.1) revealed widespread intramuscular fatty changes in the LL (including iliopsoas and glutei), chest wall and shoulder girdle muscles (including serratus anterior, subscapularis and infraspinatus).

The presence of upper motor neuron features and/or rapid progression prompted MRI of the brain and whole spine in four patients (UK1-IV.1, UK1-III.2, FR2-II.1 and FR3-II.3), which was normal in all cases.

### Molecular genetics

Heterozygous frameshift variants in *NEFH*, which segregated with the phenotype in affected family members and were inherited in an autosomal dominant fashion, were identified in all eight families through exome sequencing (UK1, UK4, FR1, FR2 and CN1) or CMT gene-specific next-generation sequencing (NGS) panels (UK2, UK3 and FR3; table 1 and figure 1). None of the variants was present in the gnomAD population database (total of 141 456 samples).^22^ Even though each family harboured one of six different frameshift variants, they all resided in the last exon of *NEFH* and shifted the reading frame to the same alternate open reading frame (ORF2, figure 1). This new frame bears a stop codon approximately 120 base pairs downstream, thus resulting in the translational elongation of the *NEFH* transcript and subsequent protein by 42–47 amino acids, depending on the size and the exact location of the variant. Therefore, from a variant interpretation perspective, these variants resulted in an almost identical gain-of-function translational effect. Since families UK1, UK2 and UK3 were found to harbour the same variant (c.3010_3011delGA) and originated from within the UK, we carried out a haplotype analysis and confirmed that they did not share the same haplotype, and hence there was no founder effect. This variant as well as the one harboured by family FR1 (c.3008_3009delAA) reside in the same homopolymer stretch (AAAAAGA) which may represent a mutational hotspot for the gene.

## Discussion

This study, undertaken on a large CMT2CC cohort, explores in depth the atypical phenotype and molecular genetics of this CMT subtype caused by specific variants in *NEFH*. This will enable more informed discussions between clinicians and patients on the natural history and prognosis of the disease, and aid the interpretation of NGS-derived novel variants in *NEFH* in the context of the observed specific phenotype.

Affected individuals display a motor-predominant sensorimotor axonal neuropathy particularly affecting the LL. Patients reported that the onset of symptoms was usually in early to mid-adulthood, with early involvement of proximal muscles (especially the iliopsoas and glutei muscles). This feature, in conjunction with evidence from the clinical assessments, confirm that the majority of CMT2CC cases are characterised by non-length-dependent weakness, unlike what is observed in the classic CMT phenotype. Since proximal muscle weakness is more commonly seen with myopathies and acquired demyelinating inflammatory neuropathies, a heightened clinical suspicion for this CMT subtype is essential. Moreover, histological analysis from nerve and muscle tissue biopsies obtained from six patients enrolled in this study (UK1-IV.4, FR1-II.1, FR1-III.3, FR1-III.4, FR1-III.7 and FR2-II.1)^19 20^ did not reveal myopathic features. Furthermore, NEFH is not expressed in muscle. This observation also concurs with neurophysiological evidence of non-length-dependent denervation seen in most patients, thus indicating that the non-length-dependent weakness that these patients display is purely neurogenic. Although some of the patients in two families (UK1 and FR1) had subtle upper motor neuron signs including brisk reflexes and extensor plantars, the weakness was not considered to be pyramidal in any of the patients.

Another striking feature of the disease is the relatively short interval from the onset of symptoms to the loss of independent ambulation (24.1±10.9 years (SD)) as illustrated by the disease progression graph in figure 3. This is most likely due to the non-length-dependent nature of the neuropathy and the early manifestation of proximal weakness. Approximately half of the symptomatic patients in this study (16/30, 53%) use a wheelchair or mobility scooter for at least part of their outdoor activities. However, for patients with CMT2CC, the lifetime risk of needing a wheelchair is likely to be greater than 53% as this is a cross-sectional study, and seven patients are still under the age of 50 years. The CMTESv2 is a clinical score designed to measure length-dependent sensory and motor impairment in CMT.^23 24^ Therefore, it was not a surprise that it does not correlate (Spearman’s r, p=0.094) with the non-length progression observed in CMT2CC.

The early involvement of muscles of the posterior compartment of the calf, including gastrocnemius, soleus and peroneus longus, resulting in early ankle plantarflexion weakness, is another unusual feature of the phenotype. This was obvious either by history (such as inability to stand on toes) or by examination of the relevant muscles and/or relative sparing of the anterior tibialis muscles on neuromuscular MRI. Even though early ankle plantarflexion weakness is rarely seen in CMT, it is nonetheless associated with specific motor-predominant subtypes, including those caused by variants in genes coding heat shock proteins, such as *HSPB1* and *HSPB8*.^25–27^

All families in this study harboured frameshift variants in the last exon of the *NEFH* gene, thus enabling the resulting aberrant transcript to escape nonsense-mediated decay and resulting in translation of the 3′-UTR. Two of the six variants (c.3010_3011delGA and c.3008_3009delAA) accounted for the genetic diagnosis in half of the families. The apparent higher prevalence of these variants in our cohort may be linked to their location within the same homopolymer stretch (AAAAAGA), since short genomic sequences such as this one are known to influence the rate of development of de novo mutations.^28^ From a variant interpretation perspective, we highlight the importance of the variant type (frameshift) and the almost identical gain-of-function translational effect they produce. All variants were absent from the gnomAD population database,^22^ which makes plausible the attribution of pathogenicity from an epidemiological perspective. Even by considering the more general toxic gain-of-function translational effect, only one such variant, which would elongate the protein by 43 amino acids, is present once (out of 250 682 alleles) in gnomAD (p.Glu995GlyfsTer68), which would respect the maximum credible population allele frequency for pathogenic variants in CMT genes as previously published.^29^ This is especially true for adult-onset CMT, including this subtype, whereby presymptomatic carriers of pathogenic variants may be enrolled in large population databases. The unique genetic features of CMT2CC (unique at least within CMT) can be used for future variant curation exercises involving indels in the last exon of *NEFH* that cause a frameshift to the same alternate reading frame. This of course should also be borne in mind when investigators encounter missense variation in the same locus, which has not been associated with CMT. Furthermore, this domain of the *NEFH* gene does not show a statistically significant missense constraint metric,^30 31^ strengthening the notion that missense variants in the last exon of *NEFH* should not be assumed to be pathogenic, unless robust genetic, bioinformatic and functional/experimental evidence suggests otherwise. Previous studies have associated in-frame deletion variants in the NEFH KSP repeat motif with ALS^10 11^; however, with the benefit of today’s large population databases of genetic variants such as gnomAD, it is obvious that such in-frame deletions in the KSP repeat motif (amino acid residues 525–832, figure 1) are frequently observed in the general population. Therefore, given the rare prevalence of ALS, epidemiologically, it would not be plausible for these variants to be causal for ALS, but they may still contribute as a polygenic risk factor.

Following the original brief clinical reports of families with CMT2CC, there have been three further reports of multigenerational families with CMT2CC and a cosegregating heterozygous frameshift variant in the last exon of *NEFH*.^32–34^ In all families, affected family members had an age of onset of symptoms in early to mid-adulthood, and a proportion displayed a non-length-dependent neuropathy with significant chronic denervation changes. Furthermore, all three families harboured heterozygous frameshift *NEFH* variants which shifted the reading frame to the same alternate one described in this study, further strengthening the association between this atypical phenotype and unique genotype.

In silico tools predict that the 3′-UTR of *NEFH* is amyloidogenic when translated.^20 21^ Furthermore, previous experimental studies with plasmid constructs that expressed the amyloidogenic 3′-UTR of the *NEFH* gene confirmed that the mutant NEFH protein forms cytoplasmic aggresomes in vivo in different cell lines,^19 20 32^ zebrafish motor neurons,^20 32^ mouse primary motor neurons and in the spinal cord of chick embryos.^19^ In the majority of the cellular models, the NEFH aggregates were perinuclear, and this is not a surprise given the destiny of NEFH proteins to coassemble locally following translation in the perikaryon and before being transported through the axon. Given the important role the phosphorylated region in the NEFH tail domain plays in various neuronal functions, including interfilament cross-bridging, axonal radial growth and axonal transport of NFs, the production of a mutant NEFH protein with an aberrant tail domain may also adversely affect the integrity of the cytoskeletal network and the neuron as a whole, making neurons more susceptible to the effect of NEFH protein aggregates.

Existing evidence suggests that accumulations of intermediate filaments, including NFs, occur in the proximal axons of lower motor neurons at a higher prevalence in ALS compared with controls,^35^ and therefore the hypothesised formation of NEFH aggregates in the proximal neuron may cause a neuronopathy and explain the non-length-dependent neuropathy and early ankle plantarflexion weakness observed in CMT2CC. Unfortunately, superficial peroneal nerve biopsies from three affected individuals described here (family FR1) and in a previously published report of CMT2CC^33^ were not examined under electron microscopy for any cytoskeletal abnormalities.

NEFM and NEFH subunits, alone or in combination, are unable to assemble in the absence of NEFL, but there is evidence that the latter can self-assemble when expressed alone in vivo.^36–38^ These observations likely reflect the critical importance of NEFL over NEFH in the assembly of the neuronal cytoskeleton and may explain the higher prevalence of *NEFL*-related CMT2E in CMT cohorts compared with the much rarer *NEFH*-related CMT2CC and an absent, so far, *NEFM*-related CMT phenotype.^39 40^ The *NEFH* variants identified in the eight probands in this study were among 2494 patients with CMT screened at the three study centres since 2016, confirming the rarity of *NEFH* variants in CMT.

In conclusion, patients with autosomal dominant CMT2CC present in adulthood with a motor-predominant non-length-dependent neuropathy, which progresses faster than expected for classical CMT with at least half of the patients losing independent ambulation by the seventh decade of life. The unique genetic pathomechanism of the disease may open potential avenues for the development of rational genetic therapies.

## Data Availability

Data are available upon reasonable request. All data relevant to the study are included in the article or uploaded as supplemental information.
