# Supplementary material for: Charcot-Marie-Tooth disease type 2CC due to NEFH variants causes a progressive, non-length-dependent, motor-predominant phenotype
Source: J Neurol Neurosurg Psychiatry. 2021 Sep 13;93(1):48–56. doi: 10.1136/jnnp-2021-327186 (PMC8685631; doi:10.1136/jnnp-2021-327186)
Supplement: Supplementary data [file jnnp-2021-327186supp001.pdf]

Charcot-Marie-Tooth disease type 2CC due to NEFH variants causes a progressive, non-length dependent, motor predominant phenotype

Supplementary table. A) Detailed clinical features from 28 phenotyped family members. Motor scores are according to the Medical Research Council (MRC) scale of muscle strength. In the column of 'LL distal muscle weakness' the two numbers, where available, represent the strength of ankle dorsiflexion and ankle plantarflexion, separated by a comma, respectively. The superscript [a] in either the symptomatology or examination indicates early ankle plantarflexion involvement and/or ankle plantarflexion weakness ≥ ankle dorsiflexion weakness. Abbreviations: AOO, age of onset; CMTESv2, Charcot-Marie-Tooth Examination Score v2.0; IVIG, intravenous immunoglobulins; LL, lower limbs; NA, not available; n/a, not applicable; OSA, obstructive sleep apnoea; UL, upper limbs; UMN, upper motor neuron.

Family - Patient	Sex	AOO (yrs)	Presenting symptoms	Proximal features / age of occurrence	Sensory sympt.	Age at assess-ment	Mobility	Muscle weakness				UMN features	CMTES v2	Additional features
								UL prox	UL distal	LL prox	LL distal			
**UK1 – IV.1**	M	4	Frequent falls; turning on ankles	Unable to stand from sitting without using arms / 21	No	31	Wheel-chair	5	4+	4	3, 1 [a]	Spastic gait in early adulthood	18	Pes cavus, clawed toes
**UK1 – III.2**	M	45	Tripping; unable to run	No	No	63	Wheel-chair	5	4	5	4, 4- [a]	Extensor plantars	14	No
**UK1 – II.1**	F	20s	Unable to stand on toes [a]	No	Numbness (mid calves)	78	Wheel-chair	5	3-4	4	1	No	20 (v1)	No
**UK2 – IV.2**	F	14	Falls	Difficulty going upstairs / 18	No	19	Indepen. walking	5	5	5	4+, 5	No	5	Achilles tendon tightness
**UK2 – III.3**	F	NA	NA	NA	NA	43	Indepen. walking	5	4	4	4	No	NA	No
**UK3 – III.2**	M	40	Legs giving way	Difficulty standing from low chair / 44	No	44	Indepen. walking	4	4	5	4+,4+ [a]	No	NA	High arches, clawed toes

Supplementary table. A) (continued).

Family - Patient	Sex	AOO (yrs)	Presenting symptoms	Proximal features / age of occurrence	Sensory sympt.	Age at assess-ment	Mobility	Muscle weakness				UMN features	CMTES v2	Additional features
								UL prox	UL distal	LL prox	LL distal			
**UK4 – IV.7**	F	35	Difficulty going upstairs	From the onset	No	53	Mobility scooter	5	5	2	4+,4+ [a]	No	8	OSA (CPAP)
**UK4 – III.1**	M	37	Walking and running difficulties; falls	Difficulty going upstairs / 50	No	75	Mobility scooter	4-5	3-4	3	0, 0	No	NA	High arches
**UK4 – III.4**	M	40	Difficulty going upstairs; unable to run	From the onset	Numbness (distal calf)	77	Wheel-chair	3	1-4	0	0, 0	No	NA	No
**UK4 – III.5**	F	35	Difficulty on stairs and going uphill	From the onset	No	73	Wheel-chair	4	4-5	0	3, 3 [a]	No	NA	No
**UK4 – IV.2**	M	12	Difficulty running	NA	Numbness (ankles)	53	Mobility scooter	4	4-5	0-3	3, 3 [a]	No	NA	No
**UK4 – IV.4**	F	13	Legs giving way	Difficulty going upstairs / 20	No	48	Mobility scooter	3-4	4+	1-3	4-, 3 [a]	No	NA	No
**UK4 – IV.8**	M	45	Legs giving way; unable to run	Difficulty going upstairs / 48	No	50	Indepen. walking	5	5	5	4+, 5-	No	NA	Jaw cramps when yawning

Supplementary table. A) (continued).

Family - Patient	Sex	AOO (yrs)	Presenting symptoms	Proximal features / age of occurrence	Sensory sympt.	Age at assess-ment	Mobility	Muscle weakness				UMN features	CMTES v2	Additional features
								UL prox	UL distal	LL prox	LL distal			
**FR1 – III.3**	F	27	Difficulty going upstairs	From the onset	No	51	Wheel-chair	5	3	2	1	No	24	Pes cavus; hypophonia
**FR1 – II.1**	F	35	Difficulty rising from floor and walking uphill	From the onset	No	70	Bed-bound	3	3	0-1	0	Brisk ankle jerks	NA	Pes cavus; Onset after pregnancy
**FR1 – II.5**	M	38	Walking difficulties	Difficulty going upstairs / 45	No	68	Indepen. walking	5	4	4	3	No	20	Pes cavus
**FR1 – III.2**	F	30	Frequent falls; unable to run	No	No	55	Indepen. walking	5	5	4	3	No	12	Pes cavus; worse after pregnancy
**FR1 – III.4**	M	30	Walking difficulties; falls	Difficulty standing up from chair / 46	No	50	Indepen. walking	5	3	4	3	No	20	Susceptible to malignant hypertherm
**FR1 – III.5**	M	43	Difficulty going upstairs	From the onset	No	49	Stick	5	4	4	3	No	17	Pes cavus
**FR1 – III.6**	F	40	Walking difficulties; falls	Difficulty going upstairs / 43	No	46	Indepen. walking	5	5	3	4	No	9	Pes cavus
**FR1 – III.7**	F	34	Unable to run	No	No	44	Stick	5	5	3	0-1	Spread knee jerks	NA	Pes cavus; onset after pregnancy
**FR1 – IV.1**	M	15	Difficult to run	No	No	23	Indepen. walking	5	5	5	4	Brisk jerks	12	Pes cavus
**FR1 – IV.5**	F	NA	Asympto-matic at 23yrs	NA	No	23	Indepen. walking	5	5	5	5	No	n/a	No
**FR1 – IV.6**	M	NA	Asympto-matic at 17yrs	NA	No	17	Indepen. walking	5	5	5	5	No	n/a	No

Supplementary table. A) (continued).

Family - Patient	Sex	AOO	Presenting symptoms	Proximal features / age of occurrence	Sensory symptoms	Age at assess-ment	Mobility	Muscle weakness				UMN features	CMTES v2	Additional features
								UL prox	UL distal	LL prox	LL distal			
**FR2 – II.1**	M	5	Difficulties in walking and jumping; falls	Difficulty going upstairs / 14	No	26	Stick; wheel-chair for long distance	3-4	4	3-4	2, 1 [a]	No	24	Pes cavus; scapular winging
**FR3 – II.3**	M	11	Difficulties in running and jumping	Difficulty going upstairs / 13	No	24	Walker; wheel-chair for long distance	4	4	0-1	0-1	No	25	Pes cavus; IVIG trial age 21 and 24 due to drastic worsening in proximal strength
**CN1 – II.2**	F	12	Difficulty standing on toes [a]	Difficulty going upstairs / 18	No	28	Stick	5	4+	3	4+	No	NA	Pes cavus; CK, 434 (nr: 32-135)
**CN1 – III.1**	F	NA	Asympto-matic at 6yrs	No	No	6	Indepen. walking	5	5	5	5	No	NA	No

Supplementary table. B) Clinical information collected by proxy on additional 5 family members. These individuals have not been assessed or genotyped but their presentation and progression suggests a likely affected status. Abbreviations: AOO, age of onset; NA, not available.

Family – Patient	Sex	AOO	Presenting symptoms	Progression	Mobility (age)
**UK1 – I.1**	M	NA	NA	Walking stick from 50s onwards	Wheelchair (70s)
**UK4 – II.2**	M	50	Walking difficulties	NA	NA
**UK4 – II.4**	M	52	Difficulty going upstairs	NA	Wheelchair (mid-70s)
**UK4 – III.3**	F	58	Unable to go upstairs	NA	Wheelchair (70)
**UK4 – IV.5**	M	45	Walking difficulties and difficulty going upstairs	NA	Walking trolley (56)
