# Supplementary material for: Charcot-Marie-Tooth disease type 2CC due to NEFH variants causes a progressive, non-length-dependent, motor-predominant phenotype
Source: J Neurol Neurosurg Psychiatry. 2021 Sep 13;93(1):48–56. doi: 10.1136/jnnp-2021-327186 (PMC8685631; doi:10.1136/jnnp-2021-327186)
Supplement: Supplementary data [file jnnp-2021-327186supp002.pdf]

Charcot-Marie-Tooth disease type 2CC due to *NEFH* variants causes a progressive, non-length dependent, motor predominant phenotype

Supplementary material

Clinical description of newly identified families with pathogenic *NEFH* variants

Family UK2

The proband from family UK2 (IV.2) had achieved normal developmental milestones and started suffering from frequent falls in her mid-teens. She complains of Achilles tendon discomfort when walking for more than 300m and since the age of 18 years, she has noticed increasing difficulty climbing stairs. She denies any sensory symptoms. On examination at the age of 19 years, she walked independently and could stand on her toes but not her heels. Motor examination was normal in the upper limbs (UL); in the lower limbs (LL) there was mild weakness distally (ankle dorsiflexion MRC 4+/5 bilaterally). There was LL areflexia and sensation was normal to pinprick, vibration and proprioception. Neurophysiology showed reduced sensory nerve action potentials (SNAPs) for age and motor responses in the LL were slightly reduced; a limited EMG of the right tibialis anterior was unremarkable.

The proband's mother (III.3) had a similar age of onset and initial severity as her daughter but had subsequently progressed significantly. On examination at the age of 43 years, she was ambulating independently with distal UL weakness (4/5) and non-length dependent weakness in the LL (proximal and distal 4/5).

Family UK3

The proband from family UK3 (III.2) was a keen dancer and in his early 30s noticed that he would suffer from leg cramps and couldn't continue dancing. At the age of 40 years, his knees started giving way and he slowly progressed such that by the age of 44 years he was unable to get off the floor or from a low chair without using his arms. He denied any sensory symptoms. On examination at the age of 44 years, he walked with bilateral foot drop and had obvious knee bobbing when standing.[1] In the UL there was mild distal weakness (FDIO and ADM 4/5). In the LL there was amyotrophy below the knees and motor examination revealed distal weakness (ankle dorsiflexion and plantarflexion 4+/5 bilaterally). All reflexes except ankles were present

and the sensory examination showed reduced pinprick to the upper calves and reduced vibration to the ankles. Neurophysiology showed absent SNAPs and reduced motor responses in the LL with MNCV in the axonal range, whereas SNAPs were severely reduced in the UL; EMG showed prominent, largely length-dependent denervation in the UL and LL.

Family UK4

The proband from family UK4 (IV.7) first noticed difficulty with walking upstairs at the age of 35 years. Her walking difficulties continued to progress over the years and by the age of 48 years she was using a walking stick when outdoors and a wheelchair for long distances; she reverted to full-time use of a mobility scooter at the age of 53 years. In her mid-40s she started experiencing difficulty with activities that required proximal upper limb strength but throughout the course of her condition she denied any sensory symptoms. On examination at the age of 53 years, she was unable to get out of the chair without using her arms and exhibited a waddling gait. There was a suggestion of proximal amyotrophy in the UL and LL. Motor examination was normal in the UL but in the LL revealed severe proximal weakness (hip flexion 2/5 bilaterally) and mild distal weakness (ankle dorsiflexion and plantarflexion 4+/5 bilaterally). LL reflexes were absent with mute plantars and sensory examination to pinprick, vibration and joint position sensation was normal. Neurophysiology showed a motor-predominant sensorimotor axonal neuropathy with prominent chronic non-length dependent denervation with a similar severity in proximal and distal muscles and occasional features of active denervation.

The proband's brother (IV.8) developed symptoms at the age of 45 years when his left leg, more than the right, started giving way and he lost the ability to run. Over the next few years, he found it particularly difficult going upstairs and suffered from falls. He also complained of jaw cramps upon yawning. On examination at the age of 50 years, he had a normal gait but difficulty in getting out of the chair. There was obvious UL and LL proximal amyotrophy, particularly in the left anterior thigh compartment, with fasciculations present. Motor examination was normal in the UL and in the LL he had mild weakness distally (ankle dorsiflexion and plantarflexion 4+/5 bilaterally). UL reflexes were absent, with reflexes patchily present at the knees and absent at the ankles. Sensory examination was normal. Neurophysiology, similar to his sister, showed a motor-predominant sensorimotor axonal neuropathy with motor

responses more affected than sensory ones and non-length dependent chronic denervation (denervation in distal and proximal muscle groups was equal).

The proband's mother (III.5) lost the ability to run and struggled walking uphill since the age of 35 years. Her LL weakness progressed resulting in falls and at the age of 60 years she noticed mild hand weakness and impaired dexterity. She started using a walking stick from the age of 40 years, a wheelchair for long distances since 60 years. On examination at the age of 73 years, she could not ambulate and needed assistance to transfer from the wheelchair to the bed. There was obvious proximal UL and proximal and distal LL symmetrical amyotrophy. Motor examination revealed mild weakness proximally and distally in the UL (shoulder abduction and external rotation 4/5 bilaterally, elbow extension 4-/5 bilaterally, FDIO and APB 4/5 bilaterally). In the LL she had severe weakness proximally with no movement in hip flexors and extensors, and distally there was ankle dorsiflexion and plantarflexion weakness at 3/5 bilaterally. She was areflexic throughout and sensory examination showed reduced pinprick sensation to both knees and reduced vibration to both ankles.

In other affected members of this family, the onset of symptoms in the form of walking and/or running difficulties, ranged from their teenage years (IV.2, IV.4) to late 30s/early 40s (III.1, III.4) and in all cases there was minimal or no sensory involvement throughout the course of disease. They all complained of progressive motor difficulties, including early features of proximal muscle weakness, and despite the variability in the age of onset they all required part or full time use of a wheelchair or mobility scooter by the time they were assessed at our centre. On examination, all members showed proximal UL weakness and severe LL weakness, with a suggestion of non-length dependency in those with a younger age of onset. Three patients showed features of early ankle plantarflexion weakness compared to ankle dorsiflexion. Deep tendon reflexes could not be elicited and there were no upper motor neuron features in any of them, and sensory examination to pinprick, vibration and proprioception revealed minimal, if any, pertinent findings. There was no evidence of vocal cord, diaphragm or respiratory muscle involvement in any family member.

Family FR3

The proband of family FR3 (II.3) initially presented at 11 years with difficulties in running and jumping. Within two years he developed difficulties in walking upstairs and his LL motor weakness continued to progress. He started using a stick for mobility

assistance at the age of 20 years and soon after required a wheelchair when covering long distances. This was due to a dramatic worsening in his proximal strength which prompted relevant investigations and two unsuccessful treatment trials with intravenous immunoglobulin (ages 21 and 24 years) for a possible inflammatory neuropathy. A CSF examination and MR imaging of the lumbosacral spine and plexuses were normal. He did not complain of any sensory involvement throughout his illness. On examination at the age of 24 years, he had a waddling gait and there was significant amyotrophy in the proximal and distal LL. Motor examination in the UL revealed mild proximal (deltoids 4/5 bilaterally) and distal weakness (intrinsic hand muscles 4/5 bilaterally). In the LL there was severe weakness proximally and distally with minimal movement in most muscle groups. He was areflexic throughout with mute plantars and sensory examination only revealed reduced vibration to the ankles. Neurophysiology revealed reduced sensory and motor responses in the UL and LL and EMG confirmed non-length dependent denervation with some occasional evidence of acute denervation.

References

1 Rossor AM, Murphy S, Reilly MM. Knee bobbing in Charcot-Marie-Tooth disease. *Pract Neurol* 2012;**12**:182–3. doi:10.1136/practneurol-2011-000167
